# Pragmatic randomised controlled trial of two brief community practice-based interventions for self-harm and suicidal ideation

**DOI:** 10.1136/bmjment-2025-301601

**Published:** 2025-05-21

**Authors:** Joanna Lockwood, Tom Goodwin, Katie Freeman, Caroline Harroe

**Affiliations:** 1 Mental Health and Clinical Neurosciences, School of Medicine, University of Nottingham, Nottingham, UK; 2 NIHR MindTech HealthTech Research Centre, University of Nottingham, Nottingham, UK; 3 Harmless, CIC, Nottingham, UK

**Keywords:** Depression, Suicide & self-harm, Child & adolescent psychiatry, Adult psychiatry

## Abstract

**Background:**

Improving preventative interventions for self-harm and suicide-related behaviour is a mental health policy priority. Existing evidence-based interventions can be lengthy, resource-heavy, difficult to access, and are not always acceptable or effective. Extending support through brief and remotely delivered interventions outside of *traditional clinical services* brings potential to expand access to timely and effective support.

**Objective:**

The primary objective is to assess the effectiveness of two brief (6 week) interventions (Integrative Therapy and Stabilisation) in reducing self-harm frequency.

**Methods:**

We evaluated data from a practice-based randomised controlled trial of hybrid Integrative Therapy and Stabilisation utilising a no-treatment control group to determine the effectiveness of each intervention targeting frequency of self-harm (primary outcome), suicidal ideation and depressive symptoms (secondary outcomes). Participants, 82 help-seeking adults with current self-harm behaviour aged 18–59 years (mean age*=*30.57, SD=12.5), received either Stabilisation (n=25) or Integrative Psychotherapy (n=25) or were assigned to a control waitlist (n=32). Six 1-hour sessions were delivered via video call in a 1:1 format. Outcome measures were completed at baseline and immediately postintervention.

**Findings:**

In comparison to waitlist controls, those receiving Stabilisation had greater reductions preintervention to postintervention in self-harm frequency, suicidal ideation and depressive symptoms. Those receiving Integrative Psychotherapy had greater reductions in self-harm frequency and suicidal ideation, but not depression symptoms, compared with waitlist.

**Conclusions:**

Interventions delivered in a service setting show promise in improving outcomes for self-harm and suicidal ideation, and to a lesser extent depression symptoms, over a 6-week period. Further evaluation and replication, including in longitudinal studies and fully randomised controlled trials, would be needed to build on these preliminary findings and extend beyond the current setting.

**Clinical implications:**

Short, remotely delivered interventions outside of traditional clinical settings may offer an effective and timely treatment option.

**WHAT IS ALREADY KNOWN ON THIS TOPIC**

- Self-harm and suicide ideation are major public health concerns, opportunities for targeted support exist within non-clinical, community-based services.

**WHAT THIS STUDY ADDS**

- This study provides preliminary evidence from a pragmatic practice-based randomised controlled trial in support of two brief interventions for self-harm, which reduced self-harm frequency in adults over a 6-week period.

**HOW THIS STUDY MIGHT AFFECT RESEARCH, PRACTICE OR POLICY**

- Scrutiny on new and non-traditional approaches to providing early, safe support for self-harm and suicidal ideation is essential given an increasing emphasis on the role of alternative settings and approaches to meet demand. Findings are preliminary and service-specific but contribute to important debates on ways to offer effective, timely and early treatment options.

## Background

 Self-harm represents a complex and often misunderstood response to psychological distress, posing a significant public health challenge in the UK. It encompasses a range of non-fatal self-injurious behaviours that individuals engage in as a maladaptive coping mechanism during times of emotional turmoil.^1^ Although not always indicative of suicidal intent, self-harm is strongly associated with an increased risk of future suicidal thoughts and behaviours, particularly when it becomes a repetitive pattern.^2 3^ Current suicide prevention strategies in England prioritise improving support for self-harm as a means of reducing suicide.^4^

Recent evidence indicates a concerning rise in self-harm rates across various demographics in the UK, with a particularly alarming increase observed among young women and girls aged 16–24.^5 6^ Hospital admissions for self-harm among young women have also seen a notable uptick, highlighting the pressing nature of this public health crisis.^7^ Although overall suicide rates in the UK remain relatively low, the surge in youth suicide and its status as the leading cause of death for individuals under 35 underscore the urgent need to address this issue.^8^ Existing responses to self-harm and suicide-related behaviours in the UK are insufficient, characterised by long wait times, a focus on crisis intervention over prevention and inadequate capacity to meet growing demands. This results in many individuals who struggle with these issues not receiving timely or appropriate support, with a significant portion not seeking or accessing formal mental health services.^9 10^

### A shift in approach

Delivering a comprehensive and effective system of care to adequately address these challenges requires moving beyond reliance on a narrow range of interventions delivered within established mental health services. This is recognised in recent national suicide prevention strategies in the UK, which emphasise earlier intervention and prevention approaches, wider involvement of community-based provision and the development of alternative, accessible and evidence-based approaches to address the evolving mental health landscape.^4^

Traditional face-to-face interventions based on cognitive-behavioural approaches such as cognitive behavioural therapy (CBT) and dialectical behaviour therapy (DBT) have shown efficacy in reducing self-harm frequency and repetition and improving mental health outcomes in adults and adolescents when compared with treatment as usual or active controls. Nonetheless, evidence is inconsistent and of low certainty.^11 12^ Standard forms of these psychological interventions are also typically lengthy and resource intensive and associated with low availability, long waitlists and poor adherence.

Efficacious shorter term and less resource-intense psychological interventions could deliver therapeutic benefit in addition to greater resource efficiency, acceptability and reach if deployed at scale.^13 14^ However, given a limited and heterogeneous evidence base (with differences in treatment, population, degree of therapist involvement and implementation context), the efficacy of brief interventions in reducing self-harm or suicide-related outcomes is not clear.^15^,^19^ A recent systematic review of brief interventions (specifically those not exceeding 240 min or four 60 min sessions) for self-injurious behaviours in young people highlighted feasible implementation across multiple specific and targeted contexts but mixed efficacy.^16^ Of note, a recent pragmatic randomised controlled trial (pRCT), which compared low-intensity outreach programmes with usual care in the prevention of suicidal behaviour in adult outpatients, found that brief online training in selected DBT skills training did not reduce risk of self-harm, and in fact may increase the risk of self-harm in some instances compared with usual care.^19^ Further studies are required to develop the evidence base for alternative forms of brief interventions as efficacious and safe treatment options.

### The current study

This study provides an independent evaluation of two distinct therapeutic interventions delivered remotely via videoconferencing: Stablisation and a hybrid Integrative Psychotherapy (IP). Both interventions are routinely offered by the third-sector organisation Harmless and seek to address the complex and multifaceted nature of self-harm and its underlying drivers.

The Stabilisation intervention emphasises a client-centred, skill-based approach rooted in DBT. This intervention prioritises immediate crisis management and emotional regulation, equipping individuals with practical DBT skills to navigate acute challenges and curb self-harm behaviours. By focusing on the ‘here and now’, stabilisation aims to provide immediate relief and foster a sense of self-efficacy, aligning with the initial stage of DBT, where behavioural Stabilisation is paramount.

The hybrid IP intervention adopts a broader, insight-oriented lens, drawing from various therapeutic modalities to facilitate a deeper understanding of the individual’s life experiences and the intricate factors contributing to their distress.^20 21^ This intervention encourages self-exploration and reflection, fostering insight into the complex interplay of past experiences, present challenges and future aspirations. By enhancing emotional regulation and self-awareness within a lifespan context, IP seeks to promote lasting change and equip individuals with healthier coping mechanisms to build resilience.

## Objective

The study’s primary objective was to evaluate data from a practice-delivered pRCT to assess the effectiveness of IP and Stabilisation in reducing self-harm frequency, utilising a no-treatment control group as a benchmark. Secondary objectives were to examine the impact of these interventions on suicidal ideation and depressive symptomatology. The study aims to provide an initial indication of treatment outcomes from service-based practice.

## Methods

### Study design

This study employed a pragmatic double-blind (participant and researcher) quasi-randomised, waitlist-controlled trial design. Interventions were not delivered concurrently but rolled out incrementally over a period of 6.5 months (from January to August 2021) reflecting the natural flow of service provision. This continuous short recruitment design is advantageous when evaluating existing services, as interventions are offered promptly on referral, minimising delays. The pRCT involved three arms: Stabilisation and the hybrid IP, both compared against a waitlist control group. Outcomes were compared after six sessions of each intervention.

### Participants

**Figure 1:**
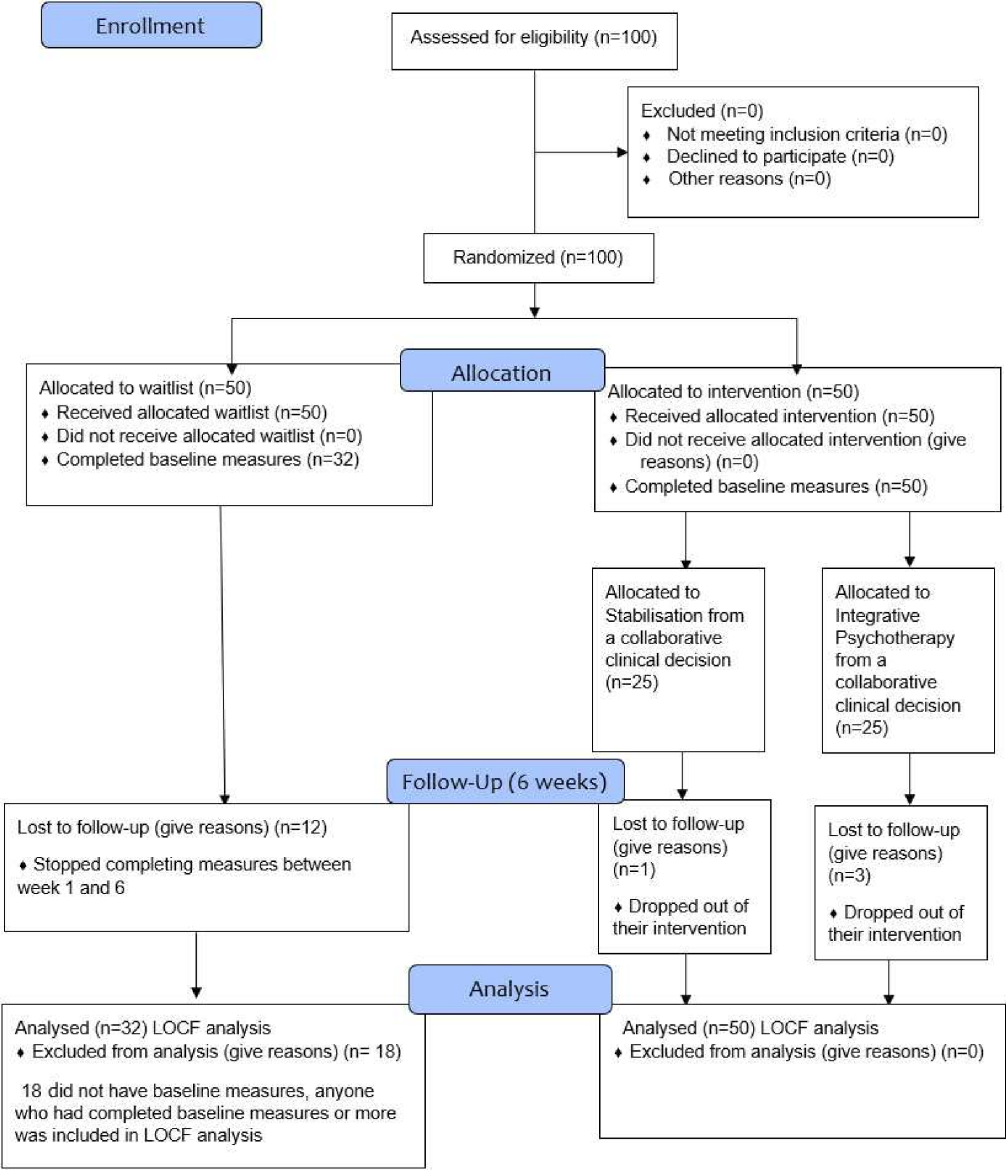
Consort flow diagram. LOCF, last observation carried forward.

The sample consisted of 82 service-users with current self-harm behaviour aged 18–59 years, mean age=30.57, SD=12.5) who had been referred to Harmless through third-sector organisations, secondary mental health services, General Practitioners, community mental health teams or self-referral (see figure 1). As standard practice at Harmless, all service users consent to the use of their anonymised data for research and service evaluation purposes. Consequently, participants remained unaware of their data’s involvement in this specific study.

Inclusion criteria mandated a baseline assessment within a week of referral, recent self-harm (one or more episodes in the preceding 2 weeks) and residency in Nottinghamshire or Leicestershire. Exclusion criteria encompassed a history of violence and being under the influence of alcohol or recreational drugs during assessment or treatment.

### Measures

*Frequency of self-harm* was assessed using a service-specific standardised self-report scale (Harmless Measure) completed with a clinician during sessions. In response to the question ‘How often have you self-harmed in the last week?’, participants were asked to mark along a 10 cm analogue scale ranging from 0 (‘not at all’) to 10 (‘very often’). The distance in centimetres from the endpoint of ‘not at all’ to the respondent’s mark on the line is converted into an integer representing self-harm frequency, rounded to the nearest centimetre. This method allows for precise quantification.

*Frequency of suicidal ideation* was recorded using the Harmless Measure, completed with a clinician in session. Participants were asked to mark along the 10 cm analogue scale in response to the question ‘To what extent have you been troubled by suicidal thoughts in the last week?’. ‘not at all’ was indicated at 0 and ‘a lot’ indicated at the 10 point. The distance in centimetres from ‘not at all’ to their digital mark was converted into an integer of suicidal ideation, rounded to the nearest centimetre.

Depressive symptoms were assessed during sessions using the standardised Patient Health Questionnaire-9 (PHQ-9). Participants were asked ‘over the last 2 weeks, how often have you been bothered by any of the following problems?’ in relation to nine topics. Answers ranged on a 4-point scale, from 0 ‘not at all’, to 1 ‘several days’, to 2 ‘more than half the days’, to 3 ‘nearly everyday’.

Primarily, data were collected online, except for two cases where telephone collection was necessary due to lack of internet access. All interventions were also conducted online, with three exceptional cases necessitating in-person delivery.

### Procedure

All participants underwent an initial 90 min baseline assessment via video call with a clinician within 7 days of their referral. During this assessment, demographic information was gathered, and the frequency of self-harm and suicidal ideation, as well as PHQ-9 outcomes, was documented. Participants were encouraged to discuss their presenting challenges, and foundational coping strategies were explored. Following this assessment, a Harmless staff members, blinded to client history, randomly allocated participants to either an intervention group (Stabilisation or IP) or the waitlist control group. This involved one staff member writing participant names on separate pieces of paper and holding one in each hand behind their back. A second staff member chose a hand at random for the intervention group and the other participant was allocated to the waitlist. Subsequently, those in the intervention groups collaboratively decided with their clinician whether to pursue IP or Stabilisation.

For IP, an assigned clinician delivered six 1-hour weekly sessions via video call. Core elements comprised a psychosocial assessment, collaborative formulation of goals, exploration of emotional needs, identification of core beliefs and thinking styles, fostering acceptance and promoting change and developing distress tolerance skills, tailored to the client’s needs and experiences.

For Stabilisation, an assigned clinical support worker delivered six 1-hour weekly sessions via video call. The primary focus was on establishing safety and emotional regulation through practical DBT skills training. Key elements included a psychosocial assessment, collaborative goal setting, problem-solving techniques, development of safer coping mechanisms and distress tolerance skills, and psychoeducation on self-harm and its impact.

Participants were asked to complete primary and secondary outcome measures weekly, up to and including the final week, session 6. Protocols for both interventions are seen in online supplemental materials S1.

Waitlist participants completed the baseline assessment via video call and then outcome measures via an online survey every 2 weeks. They were informed that they would be receiving an intervention 12 weeks postbaseline assessment. Participant responses indicating a crisis presentation necessitating intervention would be removed from the trial. There were no cases necessitating removal. Recruitment continued until sufficient participant numbers were reached.

### Treatment fidelity, clinician training and supervision

Clinical delivery and adherence to protocols were overseen by the clinical lead for the service and the service managers. Session recordings were reviewed in supervision to further consolidate fidelity. Clinical support workers delivering Stabilisation were externally trained in Stabilisation by Nottinghamshire Healthcare National Health Service Foundation Trust. IP was delivered by psychotherapists who were British Association for Counselling and Psychotherapy accredited or equivalent. Clinicians for both interventions received at minimum, monthly supervision and operated within an upper caseload limit of 20 clients per week. All clinicians had access to their own therapy.

### Statistical analysis

Three 3×2 mixed Analysis of Variance (ANOVA) tests were run in IBM SPSS statistics (V.27), with self-harm frequency, suicidal ideation and depression as dependent variables. The between-subject variable in all three analyses was treatment group (IP, Stabilisation and Waitlist) and the within-subjects variable was time (baseline (T0) and 6 weeks postintervention (T1)). Post hoc tests were planned to investigate specific between-group differences at different time points. Meta-analysis of self-harm interventions has found that active control groups (reflecting exemplary routine clinical care for self-harm) average medium effect sizes for reducing self-harm behaviour (d=0.60) and large effect sizes for reducing suicidal ideation (d=0.87) in adolescents in the absence of a comparison group. Therapeutic self-harm interventions only show very small effect sizes (d=0.13) above routine clinical care for self-harm reduction.^11^ Medium effect sizes above the waitlist control were therefore considered to indicate clinical significance, ensuring the interventions were at least as effective as routine clinical care. To detect medium effect sizes (Cohen’s f=0.25, 80% power, alpha=0.05), a sample of 42 participants was needed and the study was completed once this threshold was achieved.

### Sample characteristics

The sample consisted of 82 adults (18 male and 64 female), aged 18–59 years (M*=*30.57 years, SD=12.5). Between-group ANOVAs revealed there were no statistically significant differences between groups at baseline (see table 1). Twelve participants in the waitlist group, three in the IP group and one in the Stabilisation group did not remain in the study until the endpoint (6 weeks). There were no missing data across the multi-item depression scale. To preserve sample size and reduce attribution bias, last observation carried forward methodology was used. As such, 82 participants (who all completed baseline measures) were analysed. Data were reanalysed on a per-protocol basis with only those participants who completed six sessions, with no substantive differences.

**Table 1:** Descriptive statistics and baseline measures

Measure	Waitlist—M (SD)	I Therapy*—*M (SD)	Stabilisation*—*M (SD)	ANOVA P value^*^
Age	28.75 (11.43)	29.96 (10.40)	33.52 (15.34)	0.348
N (N-female)	32 (26)	25 (21)	25 (17)	
SH frequency	4.63 (3.00)	5.24 (3.19)	5.28 (2.97)	0.656
SI	4.97 (3.13)	4.44 (3.68)	4.96 (3.78)	0.824
Depressive symptoms†	16.92 (5.80)	16.08 (5.85)	17.92 (6.69)	0.571

* Between-group one-way ANOVAs were conducted to check for statistically significant differences at baseline.

† n=25 for waitlist for depressive symptoms to make even group sizes.

ANOVA, Analysis of Variance; M, mean; n, number; SH, self-harm; SI, suicide ideation.

## Findings

### Frequency of self-harm (primary outcome)

**Figure 2:**
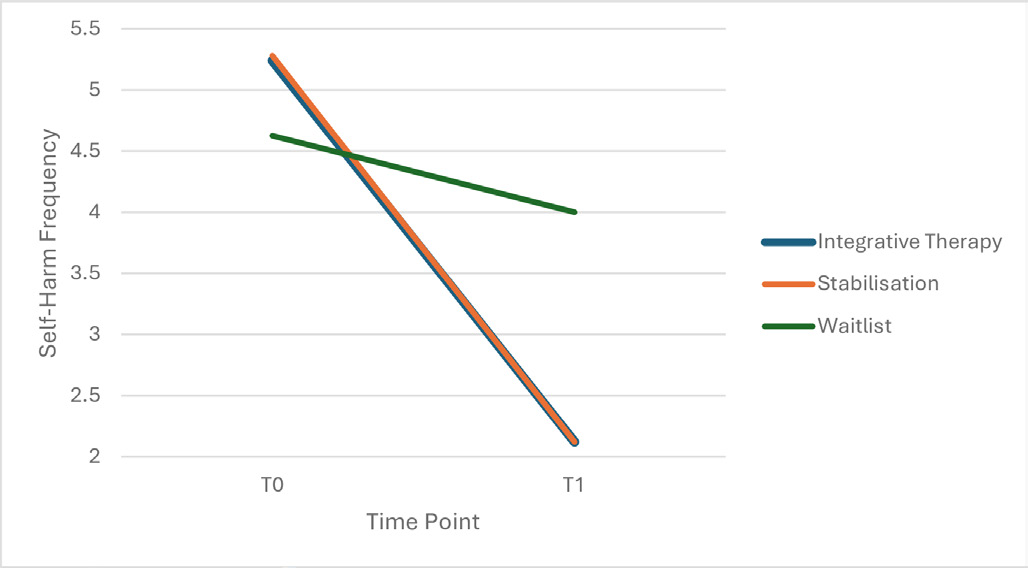
Change in self-harm frequency across interventions. Self-harm frequency captured as past week incidences using Harmless Measure.

A statistically significant interaction effect between time (T0 and T1) and group (Waitlist, IP, Stabilisation) was found (*F_2,79_*=10.847, p<0.001, *η_p_*^2^p20.215) with pairwise comparisons showing self-harm frequency significantly lower for both IP (M=2.12, SE=0.563, SD=2.22) and Stabilisation (M=2.12, SE=0.563, SD=2.50) compared with waitlist (M=4.00, SE=0.497, SD=3.39) at T1 (p<0.05; Bonferroni-corrected) with effect sizes d=0.66 and d=0.63, respectively. There was no significant difference between Stabilisation and IP at T1 (see figure 2).

### Secondary outcomes

**Figure 3:**
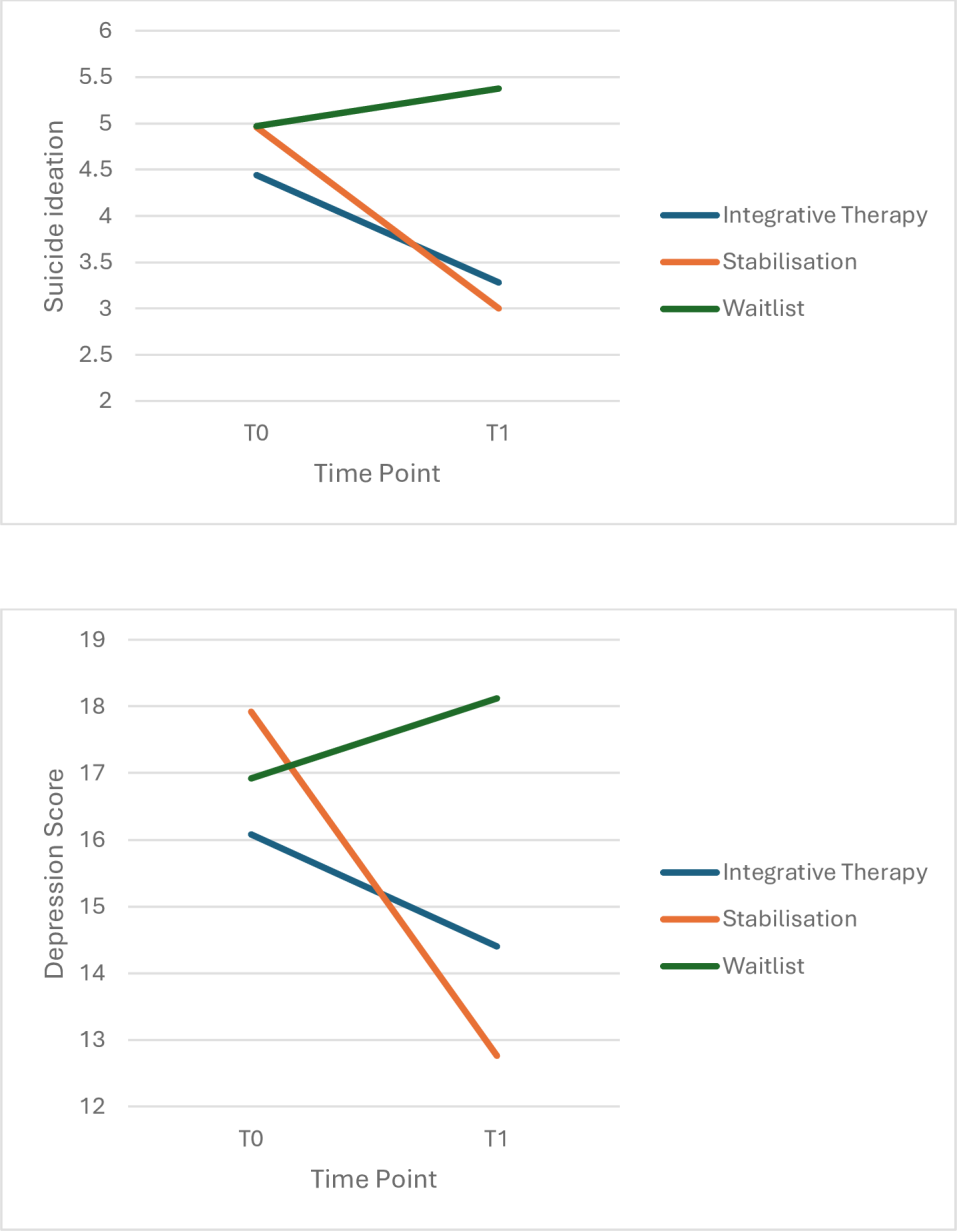
Change in secondary outcomes (suicide ideation and depression) across interventions. Suicide ideation captured as past week incidences using Harmless Measure; Depressive symptoms measured using PHQ-9 symptoms over past two weeks.

In terms of suicidal ideation, a statistically significant interaction between time and group was found (*F_2,78_*=10.847, p<0.01, *η_p_*^2^p20.140). Pairwise comparisons revealed significantly lower suicidal ideation for those in both IP (M=3.28, SE=0.630, SD=3.06) and Stabilisation groups (M=3.00, SE=0.643, SD=3.45) compared with waitlist (M=5.38, SE=0.557, SD=2.98) at T1 (both *p*s<0.05; Bonferroni-corrected), with effect sizes of d=0.70 and d=0.74, respectively. There was no significant difference between Stabilisation and IP at T1. Secondary outcomes are shown in figure 3.

In terms of depressive symptoms, a statistically significant interaction effect between Time and Group was found (*F_2,72_*=11.476, p<0.001, *η_p_*^2^p20.242). Pairwise comparisons revealed that those in the Stabilisation group (M=12.76, SE=1.384, SD=8.61) had significantly lower depressive symptoms than those in the waitlist group (M=18.120, SE=1.384, SD=6.09) at T1 (p<0.05; Bonferroni-corrected, d=0.72). There was no significant difference between IP and waitlist at T1 or between IP and stabilisation (figure 3).

## Discussion

This study offers an independent evaluation of two brief interventions, a hybrid IP and Stabilisation, targeted at self-harm reduction within a third-sector organisation. Empirical research on interventions specifically focused on self-harm reduction outside of statutory clinical services remains limited. The focus on brief, remote and third sector-delivered interventions is relevant given the increasing demand for accessible and flexible mental health support. Expanding access to mental health support beyond traditional healthcare settings is a direction supported by the NHS Long Term Plan’s emphasis on community-based and digital mental health services.^22^

### Key findings

The study provides preliminary support for the effectiveness of IP and Stabilisation in reducing self-harm frequency among help-seeking adults over the short term and suggests that both practice-based interventions offer a viable, low-intensity self-harm treatment that can be delivered remotely, which are not associated with adverse events. Participants in both intervention groups reported significantly fewer episodes of self-harm compared with those on the waitlist, demonstrating medium-to-large effect sizes. While defining a clinically meaningful reduction in self-harm frequency remains a subject of ongoing debate, the observed reduction of over 50% in both intervention groups suggests a promising short-term impact and is comparable to effect sizes achieved following longer in-person DBT interventions for individuals with borderline personality disorder.^23^

In addition, both interventions demonstrated significant reductions in suicidal ideation compared with the waitlist control group, aligning these lower intensity treatment options with the broader literature on psychosocial interventions for suicide prevention.^12 17^ The impact on depressive symptomatology was mixed. While reductions in depression symptoms were found for both interventions, these were only statistically significant for Stabilisation. It is possible that a skill-based intervention like Stabilisation can address depressive symptoms without the need for more intensive therapeutic approaches. Similar skill-based interventions targeting distress reduction and emotional regulation have shown effectiveness in reducing Post-traumatic stress disorder (PTSD) symptoms.^24 25^ Potentially, the skill-based approach of Stabilisation, even in its brief format, may have broader applications to mental health challenges. Conceivably, an insight-focused therapy (psychodynamic, emotional-focused approaches, IP) could have less immediate impact on improving mood, or even an initial worsening of symptoms, through the active process of getting in touch with pain/trauma compared with symptom-focused work (CBT, DBT, stabilisation), where practical changes could result in more immediate positive differences.

A necessary component of future examination would be to identify the active ingredients of these interventions in relation to outcome change. This is particularly important given that both interventions incorporate a blend of approaches and evidence-based principles. Of note, despite differing theoretical underpinnings, and consistent with functional accounts of self-harm,^26 27^ the focus of both Stabilisation and Integrative Therapy is on the emotional distress that often underlies self-harming behaviours. This supports targeting distress reduction as a key driver of self-harm in brief intervention development.

A study design which enables a dismantling of the various mechanisms which may underlie behaviour change, including component specifications for the current interventions, skill acquisition, cognitive restructuring, as well as the role of the therapeutic alliance, and wider contribution of the implementation context, is a useful next step. Such mechanistic research would help to tailor and optimise these approaches. It would also support potential successful translation and replication beyond this specific service setting.

The wider evidence base in relation to brief interventions for self-harm and suicide ideation is associated with mixed findings. Overall, current evidence suggests circumspection and a more nuanced dismantling of how choices relating to intervention, study population and implementation strategy are likely to impact on outcomes.

### Limitations

The study has strong ecological validity, embedded within routine clinical practice and developed in response to service identified needs. Methodological rigour is provided through a quasi-randomised, waitlist-controlled design. The pragmatic, continuous short recruitment design is advantageous when evaluating existing services, as interventions are offered promptly on referral, minimising delays. Nonetheless, the pragmatic nature of the trial raises important methodological issues. Randomisation was applied when allocating between waitlist and intervention, but thereafter a clinical decision determined intervention allocation and participants may have had a greater likelihood of benefitting from the intervention judged to be of greatest potential support, introducing bias. The service did not seek to ascertain the superiority of either intervention against each other, but against the receipt of no treatment. Nonetheless, interpretations are therefore caveated on the incorporation of clinical judgement into treatment decision-making. A fully randomised trial would be necessary to definitively establish the effectiveness of these interventions. However, the focus on the evaluation of a third-sector delivered intervention offers a timely, real-world applicability given that a large proportion of those seeking help for self-harm and suicide will not engage with statutory provision, may face long waits between referral and treatment for NHS mental health services^4 10^ and may have a treatment preference for third-sector support. Nonetheless, the generalisability of these approaches for help seekers outside of this specific service context cannot be assumed. The pragmatic nature of the study resulted in a largely female study sample that also spanned a broad range of ages (18–59 years). It is recognised that the presentation of self-harm will differ across age groups and that therapeutic treatments for self-harm differ in terms of associated cost-effectiveness for adults versus children and young people.^28 29^

The Harmless Measure, a service-based self-reported measurement tool for assessing self-harm and suicidality, has not undergone formal academic testing for reliability and validity and therefore lacks broader peer-reviewed scrutiny. The tool is not consistent with measurement approaches in comparison studies, which, for example, measure frequency in terms of discrete events. It was also not possible to assess the internal consistency of the PHQ-9 due to limited access to individual item data.

### Clinical implications

Despite these important methodological limitations, this study’s findings, while preliminary, contribute to a shifting landscape of approaches to self-harm and suicide management and prevention. If we are to challenge the traditional reliance on resource-intensive, long-term therapies and respond to calls for an expanded community-based and preventative service offer,^4 22^ then the present early scrutiny of brief remotely delivered, community-based interventions in reducing self-harm frequency and suicidal ideation is to be welcomed. Alternative and accessible approaches are needed that cater to diverse needs and circumstances, including populations who may face additional barriers to accessing traditional in-person therapies, such as those with neurodivergent conditions, limited mobility or residing in rural areas. While findings here are subjected to replication and long-term follow-up, from a clinical perspective, the significant reduction in self-harm frequency suggests that these brief interventions may be viable, first-line treatment options for individuals presenting with self-harm and could contribute to multilevel and flexible treatment options.

With demand for third-sector services increasing, and this being a preferred treatment setting for some,^10^ support offered in these settings will play an invaluable role as part of wider treatment provision, or within stepped care approaches. Providing scrutiny and evaluation of service-based treatments (as an alternative to standard therapeutic offers) and outcomes is an important first step in explicating the contribution such settings make. The present findings provide promising evidence (through a small-scale pragmatic trial) that treatment approaches within the third sector demonstrate positive effects on core therapeutic targets (self-harm frequency and suicidal ideation), are not associated with adverse outcomes, and warrant further examination.

### Future directions

A potential benefit of a briefer-form intervention for self-harm is an increased likelihood of continued engagement and session attendance and therefore receipt of a full, intended intervention dosage.^16^ Here, 92% of those receiving an intervention completed six therapeutic sessions. In the context of limited healthcare resources and an increasing demand for mental health services, the economic viability and cost-effectiveness of implementing a low-intensity intervention compared with traditional treatment approaches would need exploration. Where interventions can demonstrate comparable efficacy to more expensive, longer and intensive treatments, brief and scalable treatments should be prioritised.^30^

### Summary

This study provides early support for two brief, remotely delivered service-developed interventions, Stabilisation and IP, in reducing self-harm frequency and suicidal ideation in help-seeking adults over a 6-week period. Additionally, the Stabilisation group exhibited a notable reduction in depressive symptoms, suggesting a broader impact on mental health. Within the context of the current setting, the findings indicate clinically meaningful effects. The focus on brief and remotely deliverable interventions aligns with the priorities outlined in suicide prevention strategies and contributes to a broader discussion around the delivery of a more comprehensive and effective system of care for individuals at risk and particularly in a landscape where access to traditional therapies may be limited. Findings must be interpreted cautiously due to methodological limitations, particularly the quasi-randomisation procedure, which may have introduced bias. Overall, current evidence indicates further work to definitively establish effectiveness, maintenance and mechanisms of effect and to support the broader applicability of these interventions outside of the current service.

## Supplementary material

10.1136/bmjment-2025-301601 online supplemental file 1

## Data Availability

Data are available upon reasonable request.

## References

[1] National Institute for Health and Care Excellence (2022). Self-harm: assessment, management and preventing recurrence.

[2] Birtwistle J, Kelley R, House A (2017). Combination of self-harm methods and fatal and non-fatal repetition: A cohort study. J Affect Disord.

[3] Hawton K, Bale L, Brand F (2020). Mortality in children and adolescents following presentation to hospital after non-fatal self-harm in the Multicentre Study of Self-harm: a prospective observational cohort study. Lancet Child Adolesc Health.

[4] D.o.H.a.S. Care (2023). Suicide prevention strategy for England: 2023 to 2028.

[5] McManus S, Gunnell D (2020). Trends in mental health, non-suicidal self-harm and suicide attempts in 16-24-year old students and non-students in England, 2000-2014. Soc Psychiatry Psychiatr Epidemiol.

[6] McManus S, Gunnell D, Cooper C (2019). Prevalence of non-suicidal self-harm and service contact in England, 2000-14: repeated cross-sectional surveys of the general population. Lancet Psychiatry.

[7] Vizard T (2020). Mental health of children and young people in England, 2020.

[8] Office for National Statistics (2020). Leading causes of death, UK: 2001-2018.

[9] Appleby L (2021). The national confidential inquiry into suicide and safety in mental health.

[10] All-Party Parliamentary Group (2020). Inquiry into the provision of services for people who self-harm.

[11] Kothgassner OD, Robinson K, Goreis A (2020). Does treatment method matter? A meta-analysis of the past 20 years of research on therapeutic interventions for self-harm and suicidal ideation in adolescents. Borderline Personal Disord Emot Dysregul.

[12] Witt KG, Hetrick SE, Rajaram G (2021). Psychosocial interventions for self-harm in adults. Cochrane Database Syst Rev.

[13] Glenn CR, Esposito EC, Porter AC (2019). Evidence Base Update of Psychosocial Treatments for Self-Injurious Thoughts and Behaviors in Youth. J Clin Child Adolesc Psychol.

[14] Bettis AH, Liu RT, Walsh BW (2020). Treatments for Self-Injurious Thoughts and Behaviors in Youth: Progress and Challenges. Evid Based Pract Child Adolesc Ment Health.

[15] Kaess M, Edinger A, Fischer-Waldschmidt G (2020). Effectiveness of a brief psychotherapeutic intervention compared with treatment as usual for adolescent nonsuicidal self-injury: a single-centre, randomised controlled trial. Eur Child Adolesc Psychiatry.

[16] Dobias ML, Chen S, Fox KR (2023). Brief Interventions for Self-injurious Thoughts and Behaviors in Young People: A Systematic Review. Clin Child Fam Psychol Rev.

[17] Arshad U, Gauntlett J (2020). A Systematic Review of the Evidence Supporting Mobile- and Internet-Based Psychological Interventions For Self-Harm. Suicide Life Threat Behav.

[18] McCabe R, Garside R, Backhouse A (2018). Effectiveness of brief psychological interventions for suicidal presentations: a systematic review. BMC Psychiatry.

[19] Simon GE, Shortreed SM, Rossom RC (2022). Effect of Offering Care Management or Online Dialectical Behavior Therapy Skills Training vs Usual Care on Self-harm Among Adult Outpatients With Suicidal Ideation: A Randomized Clinical Trial. JAMA.

[20] Dawson D, Moghaddam N (2015). Formulation in action: applying psychological theory to clinical practice.

[21] Feixas G, Botella L (2004). Psychotherapy integration: reflections and contributions from a constructivist epistemology. J Psychother Integr.

[22] NHS England (2019). The NHS long term plan (NHS England).

[23] McMain SF, Guimond T, Barnhart R (2017). A randomized trial of brief dialectical behaviour therapy skills training in suicidal patients suffering from borderline disorder. Acta Psychiatr Scand.

[24] Bækkelund H, Karlsrud I, Hoffart A (2021). Stabilizing group treatment for childhood-abuse related PTSD: a randomized controlled trial. Eur J Psychotraumatol.

[25] Eichfeld C, Farrell D, Mattheß M (2019). Trauma Stabilisation as a Sole Treatment Intervention for Post-Traumatic Stress Disorder in Southeast Asia. Psychiatr Q.

[26] Edmondson AJ, Brennan CA, House AO (2016). Non-suicidal reasons for self-harm: A systematic review of self-reported accounts. J Affect Disord.

[27] Taylor PJ, Jomar K, Dhingra K (2018). A meta-analysis of the prevalence of different functions of non-suicidal self-injury. J Affect Disord.

[28] Mavranezouli I, Pelone F, Connolly R (2024). Cost-effectiveness of psychological and psychosocial interventions for adults, children and young people who have self-harmed. *BMJ Ment Health*.

[29] Troya MI, Babatunde O, Polidano K (2019). Self-harm in older adults: systematic review. Br J Psychiatry.

[30] Harris LM, Huang X, Funsch KM (2022). Efficacy of interventions for suicide and self-injury in children and adolescents: a meta-analysis. Sci Rep.

