# Supplementary material for: Pragmatic randomised controlled trial of two brief community practice-based interventions for self-harm and suicidal ideation
Source: BMJ Ment Health. 2025 May 21;28(1):e301601. doi: 10.1136/bmjment-2025-301601 (PMC12097079; doi:10.1136/bmjment-2025-301601)
Supplement: online supplemental file 1 [file bmjment-28-1-s001.docx]

Supplementary material **Intervention protocols**

Stabilisation

The first session involved a full psychosocial assessment of the client, enabling the practitioner to understand some of their life story. Session two followed, wherein the client’s needs, problems and goals were collaboratively formulated. Session three involved identifying solutions to the client’s problems and motivating the client to move towards their practical goals. The practitioners helped the clients gain an increased awareness of the drivers to their difficulties, in addition to introducing safe alternative coping methods that the client could use in place of self-harm. Session four involved reflecting on broader coping styles of the client, whilst introducing resilience building exercises and distress tolerance models to assist the client in widening their distress management strategies. Sessions five and six involved reviewing previous content to aid consolidation, with goal progress being monitored and action being promoted where necessary. Psychoeducation was then used to introduce additional coping strategies (should they be needed).

Integrative Psychotherapy

This intervention shares some similarities with stabilisation skills. Sessions one and two were almost identical, with session one involving a full psychosocial assessment, enabling the therapist to better understand the client’s life story. Session two involved the client’s needs, problems and goals being collaboratively formulated. Session three concerned understanding the pressing emotional needs of the client and placing these in the context of the self-harm in their life. Clients were also empowered to better understand the drivers of their difficulties and were introduced to strategies for developing resilience. Session four involved reviewing the broader coping strategies of the client, with a focus on core beliefs and thinking styles (as done in CBT). Distress tolerance models were also introduced at this stage. Sessions five and six involved reviewing the content so far, then the client was helped to reflect on what still needed to change and what needed to be accepted their lives to bring about some internal resolve or catharsis. This latter component of acceptance and change drew influence from DBT. Then the therapist drew upon a range of strategies (varying in accordance with the client’s needs) to help the client feel more connected internally and challenge internal styles of thinking that are contributing to their distress. The connections between thoughts, behaviours, emotions and physiology were also explored. Overall, the goal of integrative therapy was to improve distress tolerance and increase self-awareness so that the clients feel more confident and in-control of their lives. Recognising and accepting pain was a core element of this, combined with equipping the client with the knowledge and skills to make healthier life choices. It was integrative, insofar as it uses techniques from multiple therapeutic school to best meet the needs of any particular client. ‘Technical Eclecticism’, which is using proven techniques from different therapeutic approaches (Zarbo et al., 2016) is the closest variation of integrative therapy that represents the intervention.

Zarbo, C., Tasca, G. A., Cattafi, F., & Compare, A. (2016). Integrative psychotherapy works [Opinion]. *Frontiers in Psychology, 6*, Article 2021. https://doi.org/10.3389/fpsyg.2015.02021
